# ‘Recruitment, recruitment, recruitment’ – the need for more focus on retention: a qualitative study of five trials

**DOI:** 10.1186/s13063-018-2467-0

**Published:** 2018-01-29

**Authors:** Anne Daykin, Clare Clement, Carrol Gamble, Anna Kearney, Jane Blazeby, Mike Clarke, J. Athene Lane, Alison Shaw

**Affiliations:** 1 Bristol Medical School, University of Bristol, Bristol, UK; 2 Bristol Randomised Trials Collaboration (BRTC), University of Bristol, Bristol, UK; 3 MRC North West Hub for Trials Methodology Research, Institute of Translational Medicine, University of Liverpool, Liverpool, UK; 4 Collaboration and innovation in Difficult and Complex randomised controlled Trials In Invasive procedures (ConDuCT II Hub), University of Bristol, Bristol, UK; 5 Queen’s University Belfast, Belfast, Northern Ireland

**Keywords:** Retention, Qualitative study, Randomised trials, Incentives, Research nurses, Moral compass

## Abstract

**Background:**

Loss to follow-up (attrition) is a frequent problem in clinical trials and can introduce bias or reduce power. So, understanding retention issues and strategies to address these are important. As part of a multi-method project, this qualitative study aimed to explore retention strategies used by trial teams and factors which may influence strategy adoption.

**Method:**

A purposive sample of active trials was selected from the UK NIHR HTA portfolio of ongoing trials in 2014/2015. Semi-structured interviews with several trial team members from each trial and supplementary interviews with experienced trial managers explored strategies in collecting clinical outcome data and retaining participants. Interview data were analysed thematically using techniques of constant comparison.

**Results:**

Twenty-two semi-structured interviews with trial team members including chief investigators, trial managers, nurses and research administrators revealed strategies used to enhance retention. Some were recognised methods and planned from trial outset whilst others were implemented more responsively. Interviewees placed great value on fostering positive relationships with trial participants to enhance retention. However, these strategies took time which was not always appreciated by the wider trial team or funding bodies. The national focus on recruitment targets in networks posed a challenge to staff and was deemed detrimental to retention. The ‘moral compass’ of individual researchers relied on their own beliefs and values and research experience and the factors affected their confidence to pursue participant data during follow-up.

**Conclusion:**

The role of trial staff and their underlying behaviours influence retention practices and, combined with emphasis on recruitment targets, can be detrimental to motivation and retention activities. There is a need to consider how to train and support trial staff involved in retention practices and recognition of retention from funding bodies and oversight organisations.

## Background

One of the most frequent problems in clinical trials is the failure to attain completed outcome data for all recruited participants [1]. Loss to follow-up (attrition), is problematic as it can compromise study validity and lead to research waste [2]. Reduced overall sample size affects the power and effect size of the trial and bias can be introduced [3]. For instance, if characteristics or numbers of patients who drop out differ across arms, this can affect intention-to-treat analysis [4–6].

Much attention has been given to recruitment-enhancing strategies including identification and understanding of successful approaches [7]. Insight into the factors which influence decisions and behaviour underpinning these strategies has also been reported [8]. The extensive work on recruitment into trials may be relevant to other aspects of trial conduct. For example, in a synthesis of qualitative data extracted from six randomised controlled trials (RCTs), it was found that recruitment was a complex and fragile process [9]. Research nurses used their discretion over whom to inform about the RCT and to approach for recruitment, based on what they believed within their clinical or advocacy role and sometimes pre-conceived stereotypical views about groups or individuals. This research highlighted the variable enactment of a study’s protocol and that opportunities for RCT recruitment could be missed because of clinicians’, especially research nurses’ views. It was notable that many nurses were unaware of making the judgements or the rationales for doing so [9]. Thus, trial staff may face challenges managing their dual roles, faced with what they may experience as competing goals of encouraging patients to participate in a trial that may damage clinician-patient relationships [10, 11]. The above qualitative work and other research into trial recruitment [7, 11–13] indicate that the views, beliefs and experiences of trial team members are important influences on trial conduct. However, to date less attention has been given to understanding how retention strategies are developed and implemented by trial staff.

Brueton and colleagues [14] conducted a systematic review of 39 randomised studies of retention interventions nested within randomised trials. Findings identified effective strategies such as monetary incentives, but did not explore how or why strategies may work, or what participants consider to be a good incentive. Furthermore, although the eligibility criteria for the review were wider, the identified studies were also limited in that they assessed postal or electronic questionnaire response rates [15, 16]. The lack of research into retention was further highlighted by a recent survey about the methods and practices routinely used by UK clinical trials units (CTUs) to improve retention, conducted by Bower and colleagues [17]. Their survey response rate was low (38%) but their results provided examples of formal strategies used by CTUs to encourage retention such as, planning additional contacts with participants (reminders, newsletters and websites), providing flexible appointments, reducing research burden, incentives and building relationships between researchers and participants through Christmas and birthday cards. The importance of building and maintaining relationships with patients to enhance retention was highlighted.

While research on retention has identified several potential strategies, there has been little exploration of the effectiveness of such strategies, how they are used by trial researchers and how broader influences alter the effectiveness of such strategies. The ‘Losing the Losses’ study sought to address this gap by exploring reasons for failure to collect completed outcome datasets in trials. As part of a multi-method study, this qualitative component aimed to elicit how trial teams seek to retain trial participants and reasons for using retention strategies and factors which may influence their retention behaviours, to generate recommendations for retention enhancing strategies for trial teams.

## Methods

A cross-sectional qualitative study was conducted using semi-structured interviews to elicit detailed accounts from trial team members about: their current practices to maximise retention within their respective trials, reasons why these practices were employed and factors perceived to impact use of retention strategies.

### Sample and recruitment

Sampling was a two-stage process. Firstly, a purposive sample of active trials was identified from the UK National Institute for Health Research, Health Technology Assessment (NIHR HTA) portfolio of ongoing trials during 2014 (80 trials) by AK. Identification of trials involved searching the major UK funding body website and listed protocols. Sixteen trials were initially identified by AD and AS as appropriate to be approached for inclusion in the study based on an agreed sampling framework. Trials were sampled to include a range of the following: clinical topics, primary or secondary care settings, trial outcomes, length of follow-up period and procedure for collecting the trial outcome. The identified trials were discussed with the Losing the Losses study team and consensus was reached to send invitation to all to participate in qualitative study. Invitations were sent by letter to the trial chief investigators (CIs). Following expressions of interest from this sample of 16 trials, we sought to identify in the region of five trials to act as exemplar cases with a range of trial characteristics including reported retention success, clinical topics, healthcare settings (primary or secondary care) and complexity of interventions. We aimed to reach a sample of five trials which was deemed to be sufficient to address the research aim and provide enough information on retention strategies, reasons for use, and factors impacting use, across several trial types [18]. We expected to include the first five trials to agree. Secondly, within the selected ‘case study’ trials, team members were purposively sampled, using researchers’ knowledge of the trials and roles of team members within selected trials to gain a range of views on retention strategies from those having different roles within the teams. Selected members were either approached by their trial’s CI or directly by the study researcher (AD) and were invited for interview with a study information sheet which set out the aims and goals of the study. Individual written consent was given by all interviewees.

### Data collection

All interviews were conducted by AD, a skilled interviewer and experienced qualitative researcher in health research. Interviewees were not known to the study team prior to interview. Interviews were guided by a semi-structured interview schedule which allowed interviewees to expand on responses to questions and raise issues of importance from their perspective [19]. The interview topic guide covered: interviewees understanding of retention; perceptions and experiences of retention issues within the current trial, as well as strategies to enhance retention, both within the current trial and in other trials they had been involved in previously. Reasons for the use of retention strategies and factors impacting use were explored. Participants were also asked about future strategies and promising retention interventions that they would use or recommend to other trials. Interviews were carried out face-to-face or by telephone except for one participant who participated via email (this email response was concise, offering little elaboration on the questions sent). Interviews lasted between 45 and 90 min. The first part of the interview was spent developing rapport before starting questioning. Interviews were audio-recorded and transcribed verbatim for analysis purposes. Interviews were conducted between April 2014 and January 2015.

### Data analysis and rigour

Transcripts were analysed using thematic analysis [20] and methods of constant comparison [21]. Preliminary analysis was conducted by the primary researcher (AD) alongside data collection to enable early data to inform subsequent data collection. Areas of interest were flagged and pursued in more detail in subsequent interviews. Through a combination of deductive line-by-line coding based on the study aims and inductive analysis allowing for more emergent codes, an initial coding framework was developed and discussed in detail with other researchers on the team (AS, AL, AK and CG). Preliminary coding of a subset of interviews was conducted by AS, which further refined the earlier coding framework. Discrepancies in coding were identified and discussed within the research team for this study and agreement to adapt coding and restructure themes was reached. The main analysis occurred once data collection was complete using the qualitative data management software NVivo 10 (undertaken by AD and CC) [22]. Codes were grouped and refined into broader categories and higher-level recurring themes. Data were compared within and across interviews from all represented trials, according to interviewee role and trial characteristics, e.g. clinical topic. Data within themes were scrutinised for disconfirming and confirming perspectives. The analysts monitored the process until information power (sufficient range and quality of information provided by interviews to address research question) and data saturation (no new information or themes being revealed) was achieved [18, 23].

### Ethics, consent and permissions

The study was approved by the University of Bristol Faculty of Medicine and Dentistry Research Ethics Committee (13145 (10681)). All participants gave informed consent and agreed to anonymised quotes being used in publications. In presenting findings, data have been anonymised to protect confidentiality. Quotes are presented by case and role of interviewee, e.g. C4_TM (Case 4_Trial manager) or SI_STM1 (Supplementary interview_Senior trial manager 1).

## Results

Sixteen trials were invited to participate. Seven trials did not reply, one declined and two excluded themselves from the study as they felt that they had less relevant data to contribute than other trials. Of the remaining six who agreed to take part, one was unable to participate within the qualitative study timeframe and was, therefore, excluded. Within the five participating trials, 20 trial team members were approached for interview, one of whom did not respond to initial contact. Therefore, 19 trial team members from across the five trials were interviewed including, chief investigators (CIs) (x 4), trial managers (TMs) (x 5), research associates (RAs) (x 3), research fellows (RFs) (x 2) a trial administrator (TA) (x 1) and research nurses (RNs) (x 4). A further three senior trial managers (STM) who were not part of the five trial cases were approached and agreed to take part in supplementary interviews bringing the total number of interviews to 22. Senior trial managers were identified through existing team networks and recommendations from case trial teams. Twelve interviews were conducted over telephone, six were face-to-face and one was done via email. Table 1 shows the characteristics of the five trials and interviewees. Data saturation was achieved during completion of collection and analysis of the 22 interviews as agreed by the study team.

**Table 1:** Characteristics of trials and interviewees

Trial	Clinical topic	Primary or secondary care	Trial outcome	Follow-up period	How trial outcome collected	Interviewees
1	Obesity	Primary	Change in Body Mass Index (BMI) z-scores	12 months	Home visit	Chief investigator 2 x research fellow trial administrator *n =* 4
2	Renal	Secondary	Physical function	12 months	Clinic visit	Chief investigator 3 x research nurse senior trial manager trial manager *n =* 6
3	Depression and cancer	Primary and secondary	Clinical depression score	24 weeks	Home/clinic visit or telephone contact	2 x research associates research nurse trial manager *n =* 4^a^
4	Fall prevention	Primary and secondary	Rate of falls	12 months	Postal by monthly falls calendars	Chief investigator research associate trial manager *n =* 3
5	Dementia	Primary	Goal setting interview	9 months	Home visit	Chief investigator trial manager *n =* 2

^a^CI did not respond to invitation

Analysis revealed that while it was important to acknowledge the role of participants in committing to the trial and providing follow-up data; it was the behaviours of the trial team which further influenced retention. Recognised and unrecognised influences on retention are described in detail below. Recognised influences on retention were more likely to be acknowledged by teams and included (1) planned and unplanned or responsive study protocol strategies, (2) the importance of building and maintaining positive relationships with trial participants and (3) the tension caused by national oversight bodies, such as funders and research networks or, ‘the system’, placing more focus on recruitment rather than retention, to the detriment of retention. Unrecognised influences which were less overt and became more apparent to interviewees as they discussed retention and included (1) the ‘moral compass’ of individual trial team members, (2) interviewee’s level of trial experience and (3) the influence on researcher behaviour of being able to give incentives to participants.

### Recognised factors and influences on retention

#### Planned and unplanned trial processes and conduct

Interviewees identified a range of practical activities related to trial conduct which they felt could enhance retention. Most were trial process strategies and had been planned and built into the trial protocols and Standard Operating Procedures (SOPs) at the design stage and were regarded as good practice. However, some strategies were unplanned and responsive, in reaction to retention issues that emerged during the trial. Examples of planned trial conduct strategies to enhance retention included: clear and transparent data collection procedures; decreased outcome measure burden; explanation of what participation in the trial involved when seeking participants consent; presentational and communication strategies such as branding, logos, professionally printed questionnaires and trial newsletters for participants; nomination of best contact for participants and personalised participant follow-up such as a nominated trial administrator for each participant or family. Interviewees referred to these planned strategies as ‘formal’ strategies – perhaps because they were officially sanctioned or recognised within study protocols and SOPs:

‘I think the formal things are a strategy of contact. If we don’t get hold of them, sending them a letter, making sure that we have got the right phone numbers for them. Those are all formal things that we’d put in place, to make sure that we have the opportunity to get hold of parents and retain them.’ (SI_STM3)

‘We send newsletters out … I send thank-you cards after every visit.’ (C4_RA)

However, the benefits of being responsive on a case-by-case basis was also emphasised by interviewees and required a degree of flexibility with planned strategies if retention rates were low. This required staff to have the skills to think both laterally and creatively in response to ongoing retention rates:

‘It’s looking at what’s actually happening with the strategies that you’re using and just thinking laterally about what could be done to change that, or how you could implement something that maybe would improve it, and that’s what I think doesn’t happen often enough.’ (SI_STM2)

In addition to formal strategies, the interviewees also used a range of informal strategies that were not always documented in the trial paperwork. These strategies were more likely to be responsive, developed in reaction to issues that arose during the conduct of the trial and might be ‘upgraded’ to documented strategies if they proved useful as the trial progressed:

‘So, if the informal ones become useful, you can add it in as a new strategy and ask it of everyone.’ (SI_ STM2)

Whilst the interviewees could provide anecdotal suggestions of strategies they felt were useful for enhancing retention based on their prior experiences, they were aware of the lack of evidence in support of retention strategies. They gave examples of thinking about nested methodological studies within trials to obtain this evidence, such as a ‘*comparison of incentives versus no incentives*’ (C2_CI), but the majority had not been able to carry this forward due to lack of funding and resources as well as not having the methodological expertise available within the trial team to conduct such studies. However, in the view of one chief investigator, not performing nested studies was ‘*a wasted opportunity*’ (C5_CI) to enhance both the evidence base and the curricula vitae (CVs) of research staff by publishing papers on these nested studies.

#### Trial participant and team members’ interrelationships

Trial staff who interacted with participants placed great value on maintaining contact with participants and interpersonal relationships to enhance retention. Those relationships were forged using informal relational strategies; for example, by making cups of tea during trial appointments, by offering flexible appointments to suit the participants’ needs and arranging free car parking. Interviewees would also follow up participants with telephone calls if participants became ill during the trial even if the participants’ illness had nothing to do with the trial:

‘I think building a good relationship with the participant when you first meet them is very important because I think that if you’ve got a good relationship they’re more likely to stay in the study.’ (C3_TM)

However, these social relational actions required additional time which the interviewees felt was not always valued by the wider trial team and not routinely acknowledged within the funding of trials:

‘That needs to be reflected in the amount of funding that is required for research nurse time. You cannot do trials on older people where they drive up to the front desk of your clinical research centre. They nip in, you spend half an hour with them and then they bugger off again, it doesn’t work like that… I think those are important things that need to be factored in and I don’t think that some of the funding bodies get that.’ (C2_CI)

#### National focus on recruitment rather than retention

Identified as a challenge to retention in trials, interviewees felt that ‘*the system*’ (C1_CI, C2_CI) including funders and research networks placed too much emphasis on recruitment targets and insufficient attention to retention. Trial performance was often assessed on recruitment rates by monthly targets. Interviewees felt pressurised by funders to focus on recruitment, particularly at the start of the trial, for fear of the trial funding being withdrawn and the trial being stopped. Consequently, there was little focus on retention strategies at trial outset so these strategies had to be developed and implemented subsequently:

‘I do worry about that. That’s always been the case that the networks, the portfolio, all of the attention is recruit, recruit, and that’s what the financial incentives are, for the (recruitment).’ (SI_STM1)

‘We really struggle to meet the targets and there have been questions as to whether the trial would stay open and things like that. So, if you know the funding’s not going to be there, then you just focus on recruiting. I think especially at the beginning of this study, we had such a focus on recruitment, I think it was only probably 6 months in when recruitment started to kind of stabilise a bit that we thought, “Oh, God. What about retaining people? What’s our follow-up like?” We didn’t even think about it until that point.’ (C3_RA2)

Interviewees wanted an equal emphasis on recruitment and retention with a greater acknowledgment of the value of both for the scientific integrity of trials:

‘Obviously, it’s really important to keep the participants in the trial. I see the business model in here because the work and effort that goes into recruiting the patient, and that’s where all the metrics are: how many recruited? … For me, the effort that goes in has to be equal if not more to retain that participant in the trial. … Retention for me is really important, the recruitment is really important but retention definitely more so because of the effort that gets that first patient in and continue for the trial … if you don’t have the retention, you’ve got a scientific integrity issue’. (C2_STM)

The current focus of National Clinical Networks on recruitment was felt to be demotivating and some more senior trial team members allocated service support costs to aid successful retention:

‘For the portfolio, nobody’s interested in retention; they’re just interested in recruitment, but I have used the service support costs to ensure retention. So, I’ve split the service support costs so you get a certain amount for recruitment, then you get a certain amount for follow-up, or whatever; if they’re doing your notes reviews. It’s exactly like you’d pay a builder; “You do the work, then you’ll get paid. If you don’t do the work, you’re not getting paid.” … And I think that’s why, as well, we managed to get 100% (retention).’ (SI_STM3)

#### Incentives to enhance retention

There was some support for incentives targeted at trial participants for follow-up but the risk of coercion was raised:

‘I suppose you have just got to be careful about unintended consequences. Because if you are paying people to do that then there is a danger that you end up with people, not quite being coerced to stay in studies, but that is a potential problem.’ (C2_CI)

In addition, difficulties of using retention as a trial performance metric were raised with concerns that this could impact willingness of sites to take on studies with known retention challenges:

‘But also, you might get a problem that people are reluctant to become recruiting centres as there might be a significant drop out … So, if you were doing a trial with a long follow-up or you are doing a trial with a bunch of old frail people, or you are doing trials where it isn’t death and hospitalisation outcomes then you might find that people are less likely to want to become recruiting centres for those trials if you are incentivising them on retention rather than on recruitment.’ (C2_CI)

### Unrecognised influences on retention

Unrecognised influences were at first not explicitly articulated during interviews and appeared less overt to interviewees than those already discussed. As participants talked about retention, some of the more implicit and ‘unconscious’ strategies emerged and were recognised by participants during interviews, even if not thought about or acknowledged explicitly before.

#### The moral compass of individual trial team members

Interviewees seemed unaware at first of how their own ‘moral compass’ influenced retention of participants. They expressed how they often used their own beliefs and values regarding how to interact with participants, reflecting for example on how they would want their own parents to be treated, or projecting their own feelings onto a situation. At times, prioritising participants’ (projected) feelings may conflict with the pursuit of follow-up data:

‘I always say “if this was a researcher talking to my mum would I be comfortable with what they were saying?” That is my starting point, would I be happy that someone was saying this to her? If I think I wouldn’t be that happy with someone saying that then I know for me that is not right… It is your own personal value and how you would want to be treated or your family treated.’ (C5_TM)

‘I suppose it is the kind of person I am. I don’t want to hassle someone who has already got an advanced cancer and depression, I don’t feel that is right. I suppose they are my thoughts impacting on the retention I suppose.’ (C3_RA1)

Interviewees believed that the care of the participant is paramount and that coercion is unacceptable. Trial staff appeared to prioritise participants’ ‘rights’ and the ethics of their interactions with participants, which aligns with Good Clinical Practice (GCP). However, in the context of retention, their own ethics or ‘moral’ stance on acceptable pursuit of participants may clash with the goals of scientific rigour. Interviewees projected their own feelings of what is acceptable ‘chasing’ of participants and how this can be at odds with the study management’s desire for complete data collection:

‘I would be horrified to think I had coerced someone, however gentle that may be, into doing something they really didn’t want to do.’ (C2_RN2)

‘I guess in the cases like I was saying when, for instance, I was arranging the follow-ups with somebody, I knew that they didn’t want to complete this but I was told to keep going. In the other case where the woman was hanging up (the phone) on me, I knew she didn’t want me but I was told to keep going. So yes, this does happen … But the senior trial team very often just don’t understand the level of burden that the entire set of outcome measures are putting on somebody. So, that can be awkward I think … It’s hard to understand I think sometimes, the level of impairment that you’re facing with some people in terms of the length of time it’s taking and how difficult they’re finding it.’ (C3_TM)

Interviewees (usually study researchers or research nurses) with direct participant contact were continuously in conflict with the chief/principal investigator who was unaware of the potential impact of striving for complete data collection on participants:

‘I don’t know. I think maybe my boss has said like, “You should definitely try and follow them up. You should…” He’s keen to always capture data and he’ll say like, “You should go to the hospital bed and go and follow them up.” He’s kind of a bit more comfortable to push for the data a bit further, whereas I’m – maybe I’m less confident than he is. But if I feel like it might be upsetting for someone, I’ll be more likely to say, “Oh, no, it’s fine. Don’t worry. We’ll leave it there.” So I think it’s just a personal – what you feel comfortable with. Maybe I’m a bit oversensitive with that but… And I think maybe because I’ve met the patient; I’ve done their baselines; I’ve got to know them quite well. Maybe I’m just a bit more understanding of what’s going on. Whereas my boss wouldn’t have met them and he’ll just be like – he’ll just see the data, kind of thing.’ (C3_RA2)

‘I’ve spoken to (the CI) about this, and she’s – I don’t know. Maybe because she’s not actually the one doing the calls, she’s all for, “Just keep going, keep going, keep going.” I’m thinking, “Actually, to me, ethically, there comes a point…” Usually, for me, it’s after about three or four voicemails or emails. For me, that’s enough. I will not do more than that. It doesn’t feel ethically right, for me.’ (CS1_TA)

Challenges were identified for interviewees who were clinicians (e.g. counsellors or nurses), who especially felt tensions between their role of clinician and patient advocate, and trial researcher in pursuit of data. Sometimes the dual role of clinician and researcher was complementary whilst, at other times they experienced internal friction between these roles. At times, this left the clinical researchers feeling uncomfortable and demotivated to pursue data from participants:

‘Yes, it’s difficult, isn’t it? I guess that’s where I struggle, actually, because me for my way of being wouldn’t do it, but me with my trial administrator hat on says, “You’ve got to do it. We need that data.” There’s a bit of an internal wrestle with that, as well, really. Yes. Maybe I shouldn’t worry about it; just be hard-nosed and ring them up. Hmm; I’ll have to think about that one (laughter). … I guess that’s a hindrance, my background, actually, because I probably make assumptions about families, whereas if I hadn’t got my counselling training, I’d be thinking, “No; get the data, get the data.” Maybe. … To me, I thought, “I’ve got to look at the whole picture.” Yes, put like that: don’t have a counsellor being a trial administrator. It doesn’t work, really, because I think, “Well, you’re going through enough at the moment. You don’t want me saying ‘but can I…? Can I…? Can I…?’” No; it feels inappropriate.’ (C1_TA)

‘They said that they felt that usually, their role as a nurse is always to give and to care, and when they're a research nurse, they’re taking, and they’re not used to, or comfortable in doing that.’ (SI_STM3)

#### Experience of trial team members

The influence of the level of trial experience of team members on retention practices also appeared unrecognised; for example, in participant withdrawals from the trial and the extent to which they would pursue further data collection from trial participants. More experienced interviewees emphasised different levels of withdrawal and even used different terminology such as ‘*change in status*’ (SI_STM1) or ‘*opt-out of continuing*’ (SI_STM2):

‘I mean for me, if it’s me sitting in that position and I know I shouldn’t do that or one of my family members sitting there… if I say, “really from a tablet perspective, I don’t want to take any more, to me that’s enough”. I wouldn’t want to be badgered into it anymore. So, I suppose it is from a personal perspective isn’t it?’ (C2_RN2)

They felt it was ‘*crucial*’ (SI_STM2) to be flexible and were happy to negotiate with participants to at least collect primary outcome data:

‘We try very hard to make the distinction especially if it is an intervention patient, if it was withdraw from treatment or withdraw from the trial totally. What happens is often people will want to stop doing the exercises or stop wearing the insoles but they are very happy to provide us with information and fill in the questionnaires. We make this real distinction between withdrawing from treatment and withdrawing from the study. That is one option we normally talk to people about.’ (C5_TM)

Interviewees with less experience reported having less confidence to pursue participants for outcome measure data, as they assumed the participants wanted to withdraw from all aspects of the trial and made no further contact with them. Less experienced interviewees sought advice from those they deemed more experienced and adopted their advice. Interviewees also reflected on how they learnt key retention strategies ‘on the job’:

‘Yes. I think it’s definitely experience. I’d say mainly, it’s experience. Because even in the last year, I’ve got more confident about asking people and just kind of testing my beliefs or whatever. I’ve tested whether I could push for that data and ask people to do a little bit more and the end result has been okay; like no one’s really minded. So that’s made me confident … But also, like I said, we’ve had supervision and just speaking to someone who is really experienced and who at the same time, I think is a really a understanding – she’s a really experienced research nurse who I’ve got a lot of respect for and I know she’s really good with patients. Just hearing her say, “You can kind of ask if you can go to their house when they’re unwell. Or you can ask if you can do ‘this’”. Just hearing it come from her kind of made me try and get the data a bit more.’ (C3_RA2)

#### Unconscious influence of incentives

Interviewees recognised that incentives influenced retention as discussed earlier but seemed unaware that incentives may also affect their own behaviour or at least their perception of their behaviour and how they felt about their role. Incentives appeared to empower interviewees to feel more confident and comfortable maintaining contact with participants over time and more motivated to pursue acquisition of data from participants. They believed that being able to provide incentives provided them with more appeal in their interactions with participants:

‘In the (name) study, the nurses requested that it would be nice to give the children something for having taken part in the trial. They raised it with me … for the nurses, it was something nice that – they felt more comfortable then, asking for the data, if they knew that the child was going to get a little gift at the end … and I do feel that it gave the nurses a legitimate reason, in their mind, to press on with collecting the data, because they knew that the parent or the child was going to get something at the end. So if it helped them to do their job and collect the data that we needed, then it was good … the nurses were very pleased that I’d listened to them, brought it to the management group. The academics were like, a bit of a waste of money at first, and I said, “Look, I think the nurses really would like to do this, and we’ve got the money. It’s not going to do any harm.” And it was only subsequently that I came to the conclusion that actually, it’s made them realise that they feel more confident in asking, because they know that they’re going to give them something at the end.’ (SI_ STM3)

## Discussion

Interviewees in a qualitative study of five active randomised trials in the UK revealed strategies deployed by trial researchers to enhance retention. Some were methods that were well recognised, and others were new methods that had been previously unrecognised but were proactive and responsive. Key findings were the importance of building relationships between trial staff involved in retention work and the trial participants, the role of researchers’ beliefs about their responsibilities and professional values and emphasis given to recruitment means that retention is often eclipsed. Revealed within interviewees’ accounts was the influence and impact of such factors not only on trial participant behaviour but also on the views and behaviours of trial team members. This work has highlighted the importance of recognising the role of trial staff within retention practices and how current emphasis on recruitment targets can be detrimental to motivation and retention activities. There is a need to do more work to understand and consider how to support staff involved in retention practices through training, support and additional resources from the wider trial team, funding bodies and oversight organisations.

One key recognised retention strategy was incentives, either gifts or monetary. Evidence suggests that monetary incentives increase postal and electronic questionnaire response [14] possibly because they lead to more effort and higher performance from the recipient [15, 16]. Our data suggests that incentives may additionally affect the trial team member behaviour in that they feel more confident and comfortable maintaining contact with participants and more motivated to pursue the acquisition of data. This finding interestingly shows how qualitative data from interviews can support and further illuminate data from questionnaires. In addition, presenting incentives helped trial staff to feel that they had more appeal in their interactions with participants. Our study, therefore, supports the proposal that the incentives within trials should be prioritised in the funding of further methodological research to improve recruitment and retention [17] and to explore additionally why they may be effective.

Our qualitative data suggests that the trial researchers’ moral compass could compliment, but occasionally be at odds with, the overall study aim for complete data collection. There are examples of researcher’s views and beliefs affecting recruitment [11] and we identified how interviewees reflect on how they would like themselves or their own parents to be treated as a barometer of acceptable moral research practice. This ‘moral compass’ needs to be acknowledged and understood more by trial teams, with greater appreciation that those in direct contact relationships with participants may experience difficulties in dealing with recruitment pressures from trial oversight and management groups. It also needs to be noted that even when the study being conducted is a randomised trial, where bias and subjective factors which may influence differences in outcomes between the arms are reduced, personal, subjective factors still play a role in retention. These factors if not accounted for can directly affect outcomes and validity of the trial. Additionally, as shown in studies in recruitment [8, 9], trial researchers who are also clinicians may need additional support from clinical as well as research colleagues to help them manage their complex dual roles. This study showed that those with less trial experience struggled with pursuing trial data. Due to lack of experience they may be unable to use discretionary judgement and will use standard procedures to guide their task performance [21]. Support for novice researchers or those new to the clinical/researcher role may require additional support from more experienced team members. By making explicit in trial protocols the lengths that are considered acceptable to collect follow-up data as well as explicit distinctions between discontinuing treatment and discontinuing follow-up can be helpful to those on the ground. Stating specific stop criteria and supporting novice researchers would also contribute to less research waste and increasing the value gleaned from trials [2]. It may also be beneficial for trial staff to engage in a form of reflexive practice, whereby individuals engage with challenging personal beliefs and assumptions to improve professional and personal practice [24]. Such practices may be beneficial to recognise one’s own moral compass and the effect that this may be having on retention practice and should be explored within training of those working in clinical trials.

This study has highlighted that strategies tended to be implemented without an appropriate evidence base to support their effectiveness but were based on prior experience or anecdotal accounts were accepted. Embedded methodological studies were welcomed in terms of the evidence that they could provide as well as boost staff development but, due to a lack of time, money and resources it was not always possible to conduct such studies. There was also a lack of methodological expertise available within the trial teams to design and carry out methodological studies. Studies Within a Trial (SWAT) are becoming more common [25]; however, providing a robust evidence base for retention in trials through nested methodological studies needs to be explored further. For such studies to become routine, further recognition of their value, additional resources and more methodology researchers are needed.

This study has been conducted in the UK, a high-income [26] and well-resourced country but the findings may also translate to trial conduct in other less affluent countries. Social relationships between researchers and trial participants have been identified as a key factor in initial and continued trial engagement in lower- and middle-income (LMIC) countries [27]. Participant’s perceptions of a trial are influenced by interpersonal interactions and relationships with trial staff. Those with more positive encounters and social relationships are more likely to view the trial more positively and take part. However, concerns have been expressed that social relationships between those conducting and participating in research can be taken advantage of and reports have been made of how such relationships have been used to influence coercion of trial participants [28]. This can lead to unethical practice and place researchers in an uncomfortable position which, as this study found, can adversely affect retention. Motivation to take part and continue in the trial has also been shown to be influenced by appropriate remuneration and incentives in LMICs. Therefore, our findings are helpful when considering the design and conduct of trials outside of the UK and in less affluent countries.

### Strengths and limitations

This qualitative study has examined the retention practices of five trials with 22 interviewees which has captured the complexity and context of staff beliefs and resulting behaviours when pursuing follow-up data from patients in trials. Findings are based on first-hand accounts of those responsible for the day-to-day running of trials and their retention practices. Interviews allowed participants to flexibly explore their views, opinions and experiences which revealed ‘hidden’ influences and meanings integral to retention behaviours.

However, the five trials in our study are not representative of all randomised trials and were funded by one UK funding body. Trials funded through other sources may face different retention issues and pressures, particularly those who place differing emphasis on recruitment and retention, but it is likely that results are transferable across any funder. Since our study was cross-sectional we were unable to ascertain whether, and how, the retention challenges were later resolved by the trial teams and follow-up work involving observation of trial researchers’ behaviours would be of benefit. It is also possible that trials which are conducted in different health/disease areas may face different retention issues than those captured within this study. For example, telemonitoring in diabetes or chronic heart disease whereby data are automatically collected, and which may require less face-to-face contact, may benefit from increased human interactions to retain interest in the study.

There has been much discussion about the benefits and limitations of different modes of interviewing including face-to-face, telephone interviews and email [29]. Limitations of telephone interviews include less opportunity to build rapport resulting in less dynamic conversation. The interviewer for this study was an experienced interviewer and spent time at the beginning of the interviews in general conversation with interviewees which built rapport. This would have negated some of the limitations attributed to telephone interviews. As reported, the one email interview was limited in its detail. However, in comparison to the volume and depth of other interview data provided to address the research question, this limitation is considered minor.

## Conclusion

Strategies deployed by trial researchers to enhance retention included recognised and unrecognised methods. These factors are underpinned by relationships with trial participants as well as researchers’ beliefs about their responsibilities and professional values and led to planned and unplanned retention practices. However, overall the pursuit of high rates of retention is constrained by an institutional emphasis on recruitment. Recognised and unrecognised retention practices can have implications for trial outcomes and trial validity and, therefore, need to be considered when designing future randomised trials.

We therefore recommend that (1) future research should explore what is perceived to be an incentive as they are not necessarily expected to be monetary and, how and why incentives work by including their effect on researcher behaviour, (2) focus be placed on embedded studies addressing the effectiveness of retention strategies within RCTs, (3) the balance of organisational emphasis on recruitment and retention be addressed, (4) acknowledgement of the moral judgements made by staff within trials is needed and time for reflection is to be encouraged and (5) additional support and training should be provided for researchers/clinicians whilst they gain trial experience.

## Abbreviations

CI: Chief investigator
CTU: Clinical trials unit
CV: Curriculum vitae
GCP: Good Clinical Practice
NIHR HTA: National Institute for Health Research Health Technology Assessment
RA: Research associate
RCT: Randomised controlled trial
RF: Research fellow
RN: Research nurse
SOPs: Standard Operating Procedures
STM: Senior trial manager
TA: Trial administrator
TM: Trial manager
